# A long non-coding RNA, PTCSC3, as a tumor suppressor and a target of miRNAs in thyroid cancer cells

**DOI:** 10.3892/etm.2013.933

**Published:** 2013-01-30

**Authors:** MIN FAN, XINYING LI, WEI JIANG, YUN HUANG, JINGDONG LI, ZHIMING WANG

**Affiliations:** 1 Department of General Surgery, Xiangya Hospital, Central South University, Changsha 410008;; 2 Department of Geriatrics, Xiangya Second Hospital, Central South University, Changsha 410011, P.R. China

**Keywords:** thyroid cancer, papillary thryoid susceptibility candidate 3, miR-574-5p

## Abstract

Papillary thyroid carcinoma susceptibility candidate 3 (PTCSC3) is a newly identified non-coding RNA, which is highly thyroid-specific. Dramatic downregulation in thyroid cancers suggests its potential roles in the occurrence and development of thyroid tumors. The present study aimed to investigate the effects of PTCSC3 on the biological features of thyroid cancer cells and to explore its possible function as a competing endogenous RNA to bind with miRNAs. Constructs containing the long non-coding RNA, PTCSC3, were transfected into various thyroid cancer cell lines (BCPAP, FTC133 and 8505C). Cell growth, cell cycle transition and apoptosis were measured by MTT assay and flow cytometry. *In silico* analysis was performed to identify the binding site of PTCSC3 for target miRNAs. Additionally, detection of putative miRNA by quantitative reverse transcription-polymerase chain reaction (RT-PCR) in thyroid cancer cells transfected with PTCSC3 was determined to confirm the interaction. Following transfection with PTCSC3, all three thyroid cancer cells originating from various pathological types of thyroid cancers demonstrated significant growth inhibition, cell cycle arrest and increased apoptosis. The top 20 miRNAs to have a potential interaction with PTCSC3 were identified, out of which miR-574-5p was selected to further confirm the inverse correlation with PTCSC3 in thyroid cancer cells *in vitro*. In the present study, PTCSC3 as a tumor suppressor was investigated as a competing endogenous RNA for miR-574-5p.

## Introduction

Thyroid cancer is the most common malignant tumor of the endocrine organs and its incidence has been steadily increasing over the past few decades (1–3). Consistent with the majority of malignant neoplasms, thyroid cancers are usually associated with specific genetic abnormalities, as well as environmental factors (4,5). Genome-wide association studies (GWAS) determined the predisposition to papillary thyroid cancer (PTC), out of which two single nucleotide polymorphisms (SNPs; rs965513 and rs944289) located on 9q22.33 and 14q13.3, respectively, were shown to have a significant association with PTC (6–9). A long non-coding RNA gene (lncRNA) named papillary thyroid carcinoma susceptibility candidate 3 (PTCSC3) is located 3.2 kb downstream of rs944289 at 14q.13.3 (10). PTCSC3 expression is strictly thyroid-specific and is dramatically downregulated in thyroid tumor tissues and thyroid cell lines.

LncRNAs are involved in a number of regulatory functions, including modulation of apoptosis and invasion, reprogramming of induced pluripotent stem cells, acting as a marker of cell fate and parental imprinting. Previously, studies identified that lncRNA functions as a competing endogenous RNA (ceRNA) for shared miRNAs (11,12). ceRNAs demonstrate a post-transcriptional regulatory role in miRNA molecule distribution on the targets. In this study, PTCSC3, as a target of miRNAs involved in thyroid cancer, was investigated using *in silico* and biological analyses.

## Materials and methods

### Construction of the expression plasmid

The cDNA of PTCSC3 was amplified from normal human thyroid tissues (surgical specimen from benign thyroid lesion). The sequences of the forward and reverse primers were 5′-GTACGGTAC CCTCCTTCAGACTTCTCAGTACTC-3′ and 5′-CGACTC GAGATTGCTACTGTGAGCATAACCTAC-3′, respectively. Subsequently, PTCSC3 was subcloned into a pcDNA3 vector (Invitrogen Life Technologies, Carlsbad, CA, USA) to create the expression plasmid for PTCSC3 and the products were confirmed by polymerase chain reaction (PCR) and sequencing.

### Cell lines and transfection

Thyroid tumor cell lines BCPAP, FTC133 and 8505C were cultured in RPMI-1640 medium supplemented with 10% calf serum, 0.1 mM non-essential amino acids, 1 mM sodium-pyruvate and 1% penicillin-streptomycin in a 37°C humidified incubator with 5% CO_2_. The thyroid cancer cell lines were assessed for PTCSC3 expression and no endogenous expression was observed. BCPAP, FTC133 and 8505 cells, which are of papillary, follicular and anaplastic cancer origin, respectively, were transfected with the PTCSC3 expression construct and the empty vector (pcDNA3) as the control, respectively, using 2 *μ*l Lipofectamine 2000 reagent (Invitrogen Life Technologies). At 24 h post-transfection, the cells were harvested and total RNA was extracted using TRIzol reagent and Ambion^®^ DNase I (Invitrogen Life Technologies). An Agilent 2100 BioAnalyzer (Agilent Technologies, Santa Clara, CA, USA) was used to assess its integrity and a high-capacity reverse transcriptase kit (Applied Biosystems, Foster City, CA, USA) was applied to produce cDNA. To confirm successful transfection, reverse transcription (RT)-PCR was performed to detect PTCSC3 expression (forward primer, 5′-TCAAACTCCAGGGCTTGAAC-3′; reverse primer, 5′-ATTACGGCTGGGTCTACCT-3′). The study was approved by the ethics committee of Xiangya Hospital, Central South University, Changsha, China.

### MTT assay and flow cytometry

To study the changes in the biological characteristics of the thyroid cancer cell lines following PTCSC3 transfection, cell proliferation was analyzed with the MTT assay and apoptosis and cell cycle with flow cytometry, as previously described (13). For each group, cells in the logarithmic phase were used in all experiments and incubated at 37°C in 5% CO_2_. For the MTT assay, cells were incubated with 0.5 mg/ml MTT for 4 h. The formazan crystals produced by the living cells in the culture were dissolved with 100 ml dimethyl sulfoxide and the absorbance [optical density (OD) value] was measured at 570 nm using a 96-well plate reader at various time points (0, 24, 48, 72 and 96 h). For flow cytometry, the prepared cells were collected and digested into single-cell suspensions using 0.25% trypsin. Then, the cells were centrifuged at 500 × g for 5 min and washed with phosphate-buffered saline (PBS; 0.01 M, pH 7.4) twice. Seventy percent ethanol stored at 4°C was used to fix the cells for 24 h before they were fully shaken and dispensed. The plates were incubated with 0.5% Triton X-100 (Sigma, St. Louis, MO, USA) and 0.05% RNase (Sigma) in 1 ml PBS at 37°C for 30 min and then centrifuged at 1,500 × g for 5 min. The cells were stained with 50 mg/ml propidium iodide (Sigma) at room temperature for 30 min and the cell number was adjusted to 1×10^6^/ml. Samples were immediately analyzed using a FACSCalibur flow cytometer (Becton-Dickinson, Mountain View, CA, USA).

### In silico analysis of miRNAs that match PTCSC3

Bioinformatic analysis was carried out based on the online software, PITA (http://genie.weizmann.ac.il/pubs/mir07/mir07_prediction.html). Briefly, the PTCSC3 sequence, NR_049735.2, was obtained from PubMed and entered into the PITA system. As PTCSC3 is a non-protein coding RNA, it is unlikely that the open reading frame (ORF) is occupied by ribosomes. We uploaded the full sequence of PTCSC3, including the 5-untranslated region (UTR), ORF and 3-UTR, into the PITA system. Only the top 20 miRNAs presenting targeting sites in PTCSC3 were selected for further analysis.

### Quantitative RT-PCR analysis of PTCSC3 and miR-574-5p expression

To investigate the expression of miR-574-5p in thyroid cancer cells following transfection with PTCSC3, a quantitative RT-PCR technique was used. Briefly, total RNA was isolated from tissues using the TRIzol reagent (Invitrogen Life Technologies) according to the manufacturer’s instructions. Quantitative real-time PCR was performed on an ABI 7300 real-time PCR system (Applied Biosystems) using SYBR-Green mix (Applied Biosystems). Relative gene expression was calculated using the ΔΔCt method, following the manufacturer’s instructions. All reactions were carried out in triplicate. The primer sequences were: glyceraldehye 3-phosphate dehydrogenase (GAPDH), 5′- GGTGATGCTGGTGCTGAGTATGT-3′ and 5′-AAGAATGGGAGTTGCTGTTGAAGTC-3′; PTCSC3, 5′-TCAAACTCCAGGGCTTGAAC-3′ and 5′-ATT ACGGCTGGGTCTACCT-3′; miR-574-5p, 5′-GGGGTG AGTGTGTGTGTG-3′ and 5′-TGCGTGTCGTGGAGTC-3′. For each plate, a dissociation curve was obtained to monitor any additional double stranded DNA. GAPDH was used as an internal control and the formula ΔΔCt, where ΔΔCt = Ct(gene) - Ct(GAPDH), was used to calculate the relative mRNA level.

### Statistical analysis

Statistical analysis was performed using SPSS 13.0 software (SPSS Inc., Chicago, IL, USA). Comparisons were performed using Student’s t-test. P<0.05 was considered to indicate a statistically significant difference.

## Results

### PTCSC3 inhibits cell growth and induces cell cycle arrest in thyroid cancer cells

**Figure 1:**
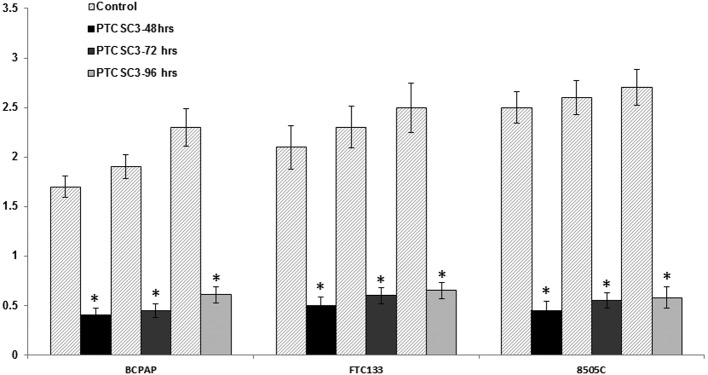
MTT assay. BCPAP, FTC133 and 8505C thyroid cancer cells (transfected with empty plasmid) had higher absorptance values at 48, 72 and 96 h compared with those transfected with PTCSC3 (^*^P<0.01). PTCSC3, papillary thyroid susceptibility candidate 3.

In order to assess the inhibitory effect of PTCSC3 on thyroid cancer cells, a cell growth assay was performed. The MTT assay revealed that absorptance values of thyroid cells (BCPAP, FTC133, 8505C) transfected with PTCSC3 at 48, 72 and 96 h were significantly lower than those transfected with the empty plasmid (P<0.01; Fig. 1). Additionally, cell cytometry revealed significant cell cycle arrest at G1/S and M/G2 phases and an increased rate of apoptosis in thyroid cancer cells (BCPAP, FTC133, 8505C) transfected with PTCSC3 compared with those transfected with the empty plasmid (P<0.01; Table I).

### Bioinformatic analysis of PTCSC3 targets

**Figure 2:**
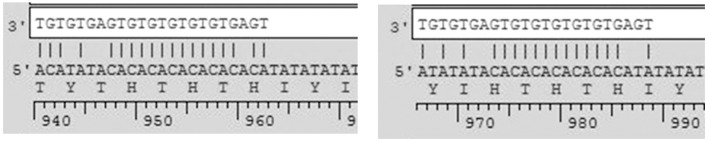
Two possible binding sites between lncRNA PTCSC3 and miR-574-5p. The upper lines are the sequence of miR-574-5p and the lower lines are the sequence of lncRNA PTCSC3. lnc, long non-coding; PTCSC3, papillary thyroid susceptibility candidate 3.

As the majority of lncRNAs share the same biogenesis mechanisms and have a similar structure with mRNAs, including the 5-UTR, ORF and 3-UTR, it is logical to assume that PTCSC3, a lncRNA, may be targeted by certain miRNAs that are upregulated in thyroid cancers. We carried out bioinformatic analyses aiming to screen miRNAs that match the 3-UTR of PTCSC3 using the online software, PITA (14). Since the ORF of mRNA is occupied by ribosomes, the majority of miRNAs target the 3-UTR of mRNAs. However, certain miRNAs target ORF of mRNAs, including the targeting of c-Myc by miR-85-3p (15). Therefore, we uploaded the full sequence of PTCSC3, including 5-UTR, ORF and 3-UTR, into the PITA system, to identify miRNAs that may target PTCSC3. As shown in Table II, twenty miRNAs have putative targeting sites in PTCSC3. Among these miRNAs, miR-574-5p is the top candidate, since it has the highest score (−30.64) according to the PITA results(14). Additionally, the matching between miR-574-5p and PTCSC3 (Fig. 2) appears perfect according to the results of miRNA-mRNA matching.

### Downregulation of miR-574-5p expression in thyroid cancer cells transfected with lnc-PTCSC3

**Figure 3:**
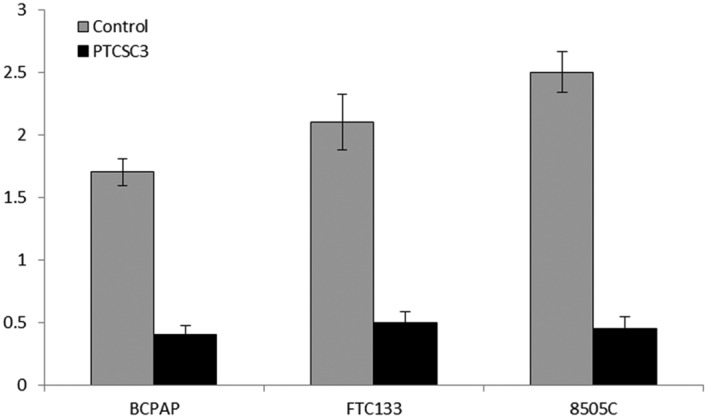
miR-574-5p expression in thyroid cell lines (BCPAP, FTC133 and 8505C) transfected with PTCSC3. PTCSC3, papillary thyroid susceptibility candidate 3.

Based on the bioinformatics study, we next confirmed this targeting in thyroid cancer cells. To investigate the possible binding of miR-574-5p with PTCSC3, miR-574-5p expression in thyroid cancer cells was determined by quantitative RT-PCR. As shown in Fig. 3, a dramatic decrease of miR-574-5p expression in all three thyroid cancer cell lines following transfection with PTCSC3 was detected (P<0.01). These data demonstrate that overexpression of PTCSC3 significantly downregulates miR-574-5p expression in thyroid cancer cells that are of papillary, follicular and anaplastic origin.

## Discussion

The newly termed PTCSC3 gene was reported to be involved in the predisposition to PTC. However, as an lncRNA, the presence and significance of PTCSC3 in thyroid cancer is undetermined. The function of individual lncRNAs may be related to epigenetic changes, action as antisense transcripts or decoys for splicing factors, as well as a competing endogeous RNA for miRNA binding (16,17). Aberrant expression of lncRNAs has a potential role in the occurrence and development of various human cancers and highlights the requirement for improved understanding of the mechanisms involved (18). In the present study, the effect of PTCSC3 on cell growth and apoptosis in thyroid cancer cells was evaluated and the possible binding of PTCSC3 with specific miRNAs was studied by bioinformatic and biological analyses.

The highly thyroid-specific expression of PTCSC3 and downregulated expression in PTC tissues and cell lines implicated its potential role in thyroid cancer. To date, only one study has investigated the biological feature changes of PTC cells following transfection with PTCSC3 (10). In the present study, three thyroid cancer cell lines of various histo-pathological origin (papillary, follicular and anaplastic thyroid cancer) were selected to study the effect of PTCSC3 on thyroid cancer originating from follicular epithelial cells. Collectively, our data demonstrate that overexpression of PTCSC3 in thyroid cancer cells inhibits cellular proliferation and induces cell cycle arrest and apoptosis, suggesting that dysfunction of PTCSC3 in thyroid cancer may be a common molecular event. It is essential to develop *in vivo* animal models with PTCSC3 expression in the thyroid to demonstrate the role of PTCSC3 in tumorigenesis in the future.

According to the ceRNA hypothesis, lncRNAs may elicit their biological activity through their ability to act as endogenous decoys for miRNAs; such activity in turn affects the distribution of miRNAs on their targets (11). Bioinformatic analysis of lncRNAs is an important method for discovering the relevant functions (19). We searched for miRNA recognition motifs in the PTCSC3 sequence and the presence of recognition sites for possible miRNAs. As shown in Table II, out of the top 20 miRNAs that were identified, miR-574-5p was selected to validate the interaction in thyroid cancer cells. To further comfirm the binding possibility of PTCSC3 with miR-574-5p, the miR-574-5p expression in various thyroid cell lines transfected with PTCSC3 was detected using quantitative RT-PCR. The results revealed that miR-574-5p expression was dramatically reduced due to overexpression of PTCSC3. The significant inverse correlation between PTCSC3 and miR-574-5p suggests that PTCSC3 acts as a competing endogenous RNA to target miRNAs and in turn regulate cell growth and apoptosis in thyroid cancer. Site-directed mutagenesis assay was performed to confirm the binding site. Although there are no expression studies regarding miR-574-5p in thyroid cancer cells, miR-574-5p was previously identified to be associated with various human cancers as oncogenic miRNA. As far as miR-574-5p is concerned, the discovered functions include proliferation and anchorage-independent growth of cancer cells in head and neck squamous cell carcinoma, chemoresistance and poor survival in patients with small-cell lung cancer, as well as colorectal cancer tumorigenesis and progression at the early stages (20–22). The present study provides information concerning the function of PTCSC3; however, further investigations are required on lncRNA PTCSC3 and its association with miRNAs in thyroid cancer. Moreover, understanding the molecular mechanisms of PTCSC3 in thyroid cancer is fundamentally important in developing new molecular markers for earlier diagnosis and novel therapeutic targets.

## Figures and Tables

**Table I:** G0/G1, S, G2/M and apoptosis rate in thyroid cancer cell lines transfected with PTCSC3.

Group	G0/G1	S	G2/M	Apoptosis
BCPAP				
Control	26.2±2.1	46.6±5.1	27.2±3.6	3.6±1.0
PTCSC3	46.8±5.2^a^	35.9±4.1^a^	17.3±2.2^a^	6.9±1.9^a^
FTC133				
Control	27.7±3.4	45.6±4.4	26.7±2.5	2.9±1.2
PTCSC3	46.4±6.7^a^	34.2±3.6^a^	19.4±1.8^a^	5.8±1.5^a^
8505C				
Control	25.7±3.2	54.2±5.3	14.2±2.1	2.1±1.1
PTCSC3	45.6±5.7^a^	40.2±4.3^a^	20.1±2.6^a^	6.3±2.3^a^

a P<0.01. Data are presented as mean ± standard deviation (%); n=6. PTCSC3, papillary thyroid susceptibility candidate 3.

**Table II:** Top 20 microRNAs as candidates for PTCSC3 targets.

Gene	microRNA	Sites	Score
PTCSC3	hsa-miR-574-5p	17	−30.64
PTCSC3	hsa-miR-1207-5p	2	−21.84
PTCSC3	hsa-miR-939	4	−21.15
PTCSC3	hsa-miR-637	4	−18.05
PTCSC3	hsa-miR-1260	3	−17.18
PTCSC3	hsa-miR-297	22	−16.66
PTCSC3	hsa-miR-1229	4	−16.04
PTCSC3	hsa-miR-920	4	−15.82
PTCSC3	hsa-miR-212	4	−15.49
PTCSC3	hsa-miR-574-3p	2	−15.48
PTCSC3	hsa-miR-1182	4	−15.4
PTCSC3	hsa-miR-1280	1	−15.04
PTCSC3	hsa-miR-885-5p	4	−14.98
PTCSC3	hsa-miR-326	3	−14.96
PTCSC3	hsa-miR-453	2	−14.2
PTCSC3	hsa-miR-511	10	−14.13
PTCSC3	hsa-miR-34c-3p	1	−13.96
PTCSC3	hsa-miR-612	3	−13.9
PTCSC3	hsa-miR-188-3p	2	−13.49
PTCSC3	hsa-miR-210	17	−13.45

PTCSC3, papillary thyroid susceptibility candidate 3; hsa, human serum albumin.
